# All-metal wideband metasurface for near-field transformation of medium-to-high gain electromagnetic sources

**DOI:** 10.1038/s41598-021-88547-3

**Published:** 2021-05-03

**Authors:** Ali Lalbakhsh, Muhammad U. Afzal, Touseef Hayat, Karu P. Esselle, Kaushik Mandal

**Affiliations:** 1 School of Engineering, Macquarie University, Sydney, Australia; 2 School of Electrical and Data Engineering, University of Technology Sydney (UTS), Sydney, NSW Australia; 3 Institute of Radio Physics and Electronics, University of Calcutta, Kolkata, India

**Keywords:** Electrical and electronic engineering, Electronic and spintronic devices

## Abstract

Electromagnetic (EM) metasurfaces are essential in a wide range of EM engineering applications, from incorporated into antenna designs to separate devices like radome. Near-field manipulators are a class of metasurfaces engineered to tailor an EM source’s radiation patterns by manipulating its near-field components. They can be made of all-dielectric, hybrid, or all-metal materials; however, simultaneously delivering a set of desired specifications by an all-metal structure is more challenging due to limitations of a substrate-less configuration. The existing near-field phase manipulators have at least one of the following limitations; expensive dielectric-based prototyping, subject to ray tracing approximation and conditions, narrowband performance, costly manufacturing, and polarization dependence. In contrast, we propose an all-metal wideband phase correcting structure (AWPCS) with none of these limitations and is designed based on the relative phase error extracted by post-processing the actual near-field distributions of any EM sources. Hence, it is applicable to any antennas, including those that cannot be accurately analyzed with ray-tracing, particularly for near-field analysis. To experimentally verify the wideband performance of the AWPCS, a shortened horn antenna with a large apex angle and a non-uniform near-field phase distribution is used as an EM source for the AWPCS. The measured results verify a significant improvement in the antenna’s aperture phase distribution in a large frequency band of 25%.

## Introduction

There has been an increasing demand of low-cost, light-weight directive antennas for micro- and millimeter wave frequency bands. Traditionally, reflector dishes and arrays of low-gain antennas, such as microstrip antennas are employed in high-gain applications^1^. The former is undesirably large for some applications and the latter needs a complex feeding network subject to loss. Lately, Resonant-Cavity Antennas (RCAs) have been investigated for high-gain applications because of their simple feed mechanism and planar configurations^2–7^. However, the conventional RCAs have a non-uniform phase distribution created by the transverse propagation of the cavity feed, which degrades their far-field radiation patterns. This near-field problem was rectified by the revelation of Phase Correcting Structures (PCSs) in 2015^8,9^, which do not obey lens theory to estimate the phase errors in the antenna aperture.

Since then, several PCSs have been proposed for near-field modifications of aperture antennas^10–16^. The first generation of PCSs was made of varying dielectric thickness, locally distributed to create sufficient localized time-delay on the antennas aperture with a spherical phase front^8,9^. Despite the significant near-field modifications achieved by them, the high prototyping cost, heavy weight and high profile are disadvantages associated with this generation of PCSs. later on, a semi-planar structure composed of different permittivites was used to avoid an undesirably high profile of the all-dielectric PCSs at a price of more complex fabrication^10^. To mitigate the limitations of all-dielectric PCSs, the second generation of PCSs was introduced, where the thick dielectric near-field manipulating regions were replaced by planar printed microwave substrates. This type of FSS was equally successful in near-field rectification of highly non-uniform apertures, while it has the advantage of having a very low profile compared to the all-dielectric PCSs.

Nevertheless, all aforementioned solutions are composed of either all-dielectric or conductive elements mounted on the expensive microwave dielectric substrates, contributing to very high prototyping cost. Additionally, it may be preferred to avoid employing such substrates in applications with harsh environments, such as space explorations^17^.

Very recently, a few all-metal phase-shifting structures have been proposed, all of which are polarization dependent and/or operate at a single frequency^15,18–23^. For example, structures proposed in^18^ and^22^ have a very limited bandwidth and can only support either circularly, or linearly polarized waves. The metasurface in^20^ only support circular polarization within two limited passbands and requires 3D metal prototyping. Although, the all-metal PCS comprised of non-resonating patch elements in^15^ can support all types of polarization, each phase-shifting element operates at a single frequency, resulting in a highly narrow-band PCS. Differently, large operational bandwidths of around 24% and 15% have been achieved in^21^ and^19^, respectively, where the former is suitable for linear polarization only and the latter achieves the required phase-shift by transforming the polarization of the incoming wave into a cross polar outgoing wave.

Reviewing near-field correcting structures, which do not rely on lens theory, suggests that while considerable near-filed enhancements have been achieved by various types PCSs, there is still no fully-metallic passive solution for a wideband polarization-insensitive near-field correction. Unlike all other PCSs, here we use resonating elements to synthesize a substrate-less free-standing PCS, named AWPCS, whose wideband operational mechanism does not rely on polarization conversion or the polarization of the incoming EM waves. Additionally, the AWPCS is low-cost, light-weight and unlike^18^ does not require any internal waveguide, so it can be developed using a range of low-cost manufacturing technologies, such as water-jet cutting, laser cutting and 2D stamping for rapid large-scale prototyping. For the proof of concept, and verifying the wideband performance of the proposed AWPCS, an electromagnetic source with a non-uniform near-field phase distribution and a sufficiently large operational frequency band is required to be used as the base antenna for the AWPCS. Here, we use a heavily shortened horn antenna for this purpose.

The rest of the paper is organized as follows: In “Shortened conical horn antenna with larger apex angle”, a shortened horn antenna with a large apex having a highly non-uniform electric near-field phase distribution is designed. In “Electromagnetic and mechanical specifications of the proposed unit cell”, the novel structure of the proposed AWPCS along with its electromagnetic and mechanical specifications are presented. “AWPCS prototyping” discusses the fabrication and the measurements of the antenna system, and “Near- and far-field results” gives a summary of the paper.

## Shortened conical horn antenna with larger apex angle

The physical parameters of a conventional conical horn fed by a waveguide is calculated using Eqs. (1) and (2) and some trigonometric relations as discussed in^24^.

1 $$\begin{aligned} G= & {} \varepsilon _{ap}\frac{4\pi A_{p} }{\lambda ^{2}} \end{aligned}$$

2 $$\begin{aligned} r= & {} \frac{\sqrt{3 l \lambda }}{2} \end{aligned}$$

In Eq. (1), *G* is the gain, $\varepsilon _{ap}$ is the aperture efficiency, $\lambda$ is the free-space wavelength at the design frequency, $A_{p}$ is the physical aperture of the antenna. In Eq. (1), *r* and *l* are the radii and length of the conical horn, respectively.

**Figure 1:**
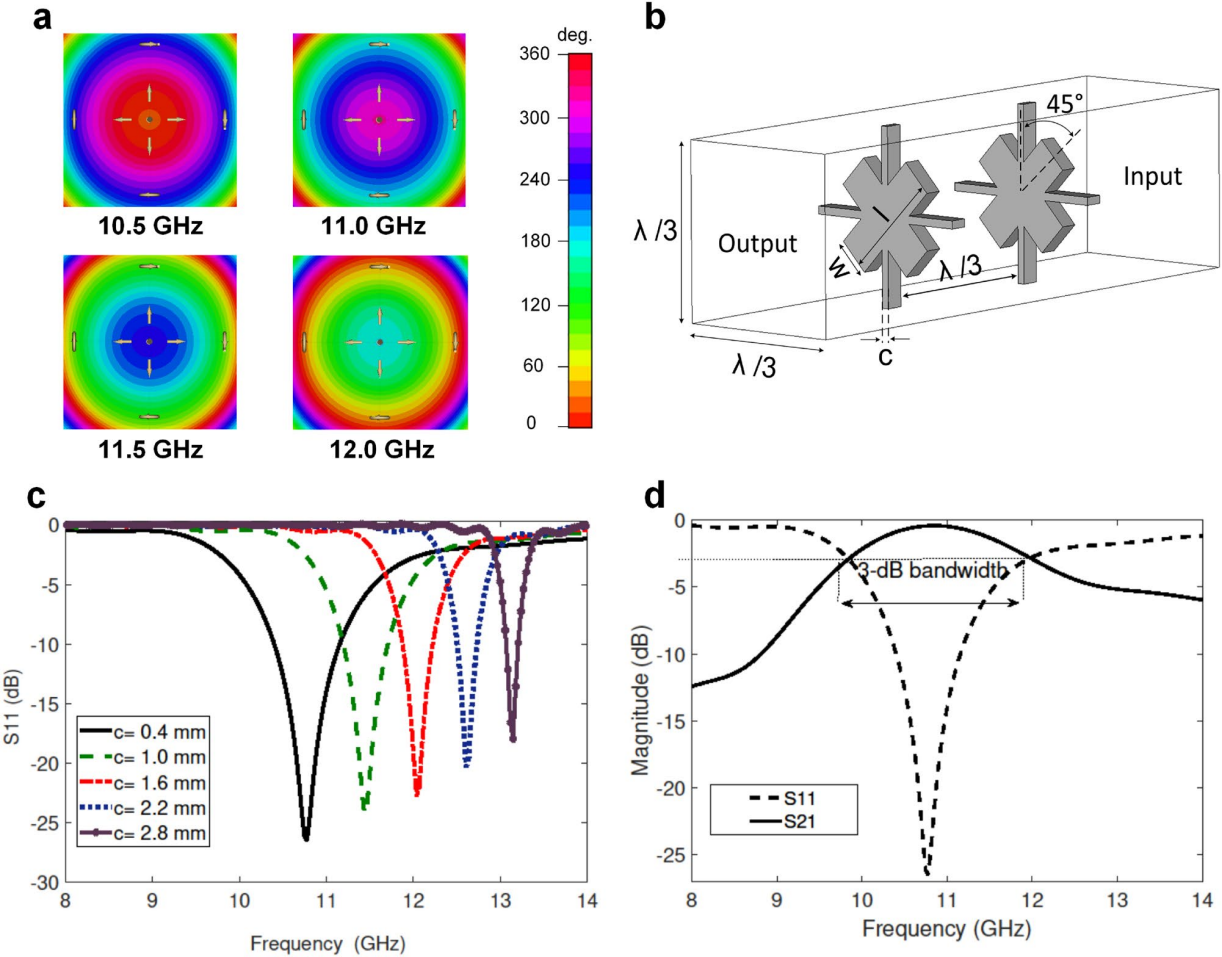
**(a)** 2D phase distribution of the shortened horn antenna on a reference surface at a distance of 15 mm from the aperture. **(b)** A perspective of the proposed fully metallic spatial phase shifter. **(c)** The magnitude of the reflection coefficients of the bandpass CSs for different widths. **(d)** Transmission magnitude of the SC (c = 0.4 mm).

In order to achieve a peak gain of 21 dB at 11 GHz, the height and radii of the conventional conical horn are calculated to be 192.7 mm and 68.9 mm, respectively. Increasing the flare angle from $17.26 ^\circ$ to $32^\circ$ and reducing the height to 70.7 mm, result in a new ad hoc horn with an aperture radii of 53 mm. The ad hoc horn has a significantly smaller volume (4.6 times volume reduction) and a severely deteriorated near-field phase distribution, resulting a peak gain of 16.05 dB. This can be seen from 2-D near-field phase distributions at 4 distinct frequencies within the desired frequency band (10.0 GHz to 12.5 GHz) in Fig. 1a. Such undesired spherical wavefronts can be rectified by the proposed AWPCS explained in the following section.

## Design of the all-metal phase correcting structure (AWPCS)

### Electromagnetic and mechanical specifications of the proposed unit cell

Over the last few years, metasurfaces have made breakthroughs in electromagnetic problems by introducing engineered microwave properties which are not achievable using conventional antennas and microwave devices^25–39^. There are a number of phase-correcting designs based on printed metasurfaces, which are bound to use dielectric substrates to support and isolate the conductive patterns on the printed layers^14,40–44^. However, the existing knowledge of such metasurfaces cannot be directly applied on a substrate-less structure. This problem is more restricting for the wideband applications, due to the intrinsic complexity associated with the design of wideband spatial phase shifters.

Hence, design of the AWPCS is not only a pure EM design problem, but mechanical restrictions need to be initially removed by some none-electromagnetically intrusive approach. Once the structure’s unconditional mechanical integrity is ensured, metallic resonators with any configurations, with or without discontinuity can be incorporated in the structure, which was otherwise not possible.

Figure 1b shows a unit-cell configuration of the proposed metallic spatial phase shifter with a unit-cell size of $\lambda /3$ following recommendations in^10,14^. As shown in Fig. 1b, each unit cell comprises of two identical metallic layers separated by an air-gap of $\lambda /3$. Each layer is composed of two pairs of orthogonal strips with an angle of $45^o$ from each other, as illustrated in Fig. 1b. In this topology, the throughout inductive strips are named Static Cross (SC), which is primarily responsible for mechanical integrity of the structure, while the diagonal dipole is called Diagonal Resonator (DR) and designed to be varied in both length and width to generate the required phase shift without upsetting the structural robustness ensured by the SCs. The thickness of metal layers is chosen to be 1 mm, so that the AWPCS can be fabricated using low-cost available technologies, and would not require any extra bonding and protecting mechanism like foam or radome.

**Figure 2:**
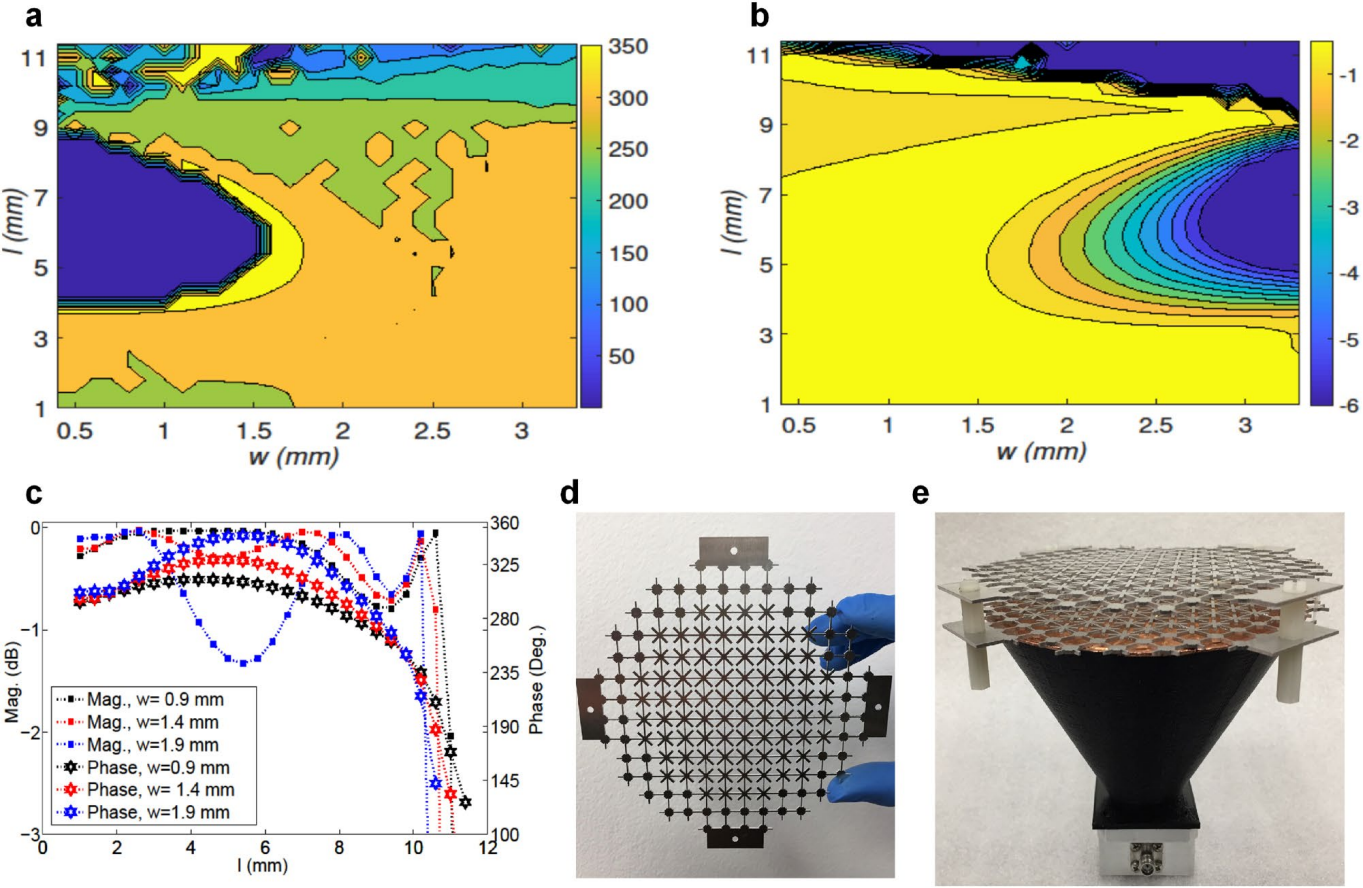
**(a)** Transmission phase of various combinations of *l* and *w* for a constant c, (c = 0.4 mm). **(b)** Transmission magnitude of various combinations of *l* and *w* for a constant c, (c = 0.4 mm). **(c)** Transmission coefficients of the selected unit cells for varying size of DRs. **(d)** One layer of the AWPCS fabricated using waterjet cutting with abrasive garnet powder. **(e)** Fabricated AWPCS placed on the shortened horn antenna. The two concentric layers of the AWPCS is separated by 4 nylon spacers.

To achieve wideband performance of the AWPCS, the SCs in the absence of DRs need to have a large passband, covering the desired operation frequency of the AWPCS. This can be achieved if the resonance condition is met somewhere in the operating frequency band. In this design, the presence of two inductive strips in SCs and the capacitive effects created by the stacked air-transmission line create a resonance condition which can be tuned at 11 GHz using a parametric analysis, as illustrated in Fig. 1c. According to this analysis, a transmission pole occurs at 10.8 GHz for the case of $c=0.4$ mm, and shifts to higher frequencies when *c* increases. As a result, a large 3-dB transmission band with a fractional bandwidth of 21% centered at 11 GHz is realized and shown in Fig. 1d.

Once the design of the SC is finalized and the mechanical integrity of the structure is ensured, the required phase shifts can be achieved for different dimensions of DRs. The transmission magnitude and phase of each cell were numerically predicted through a parametric study of the unit cell shown in Fig. 1b, where *l* and *w* varied and SC remained unchanged. In this study, *l* was swept from 0.4 to 3.4 mm with a step size of 0.1 mm, and *w* varied from 1.0 to 11.4 mm with an interval of 0.4 mm. The minimum, maximum and the step size values in this analysis are determined considering the tolerance limits of the fabrication process and the sensitivity of the parameters. The optimal sizes of *l* and *w* can be determined using surface plots shown in Fig. 2a,b, where the magnitude and the phase of transmission coefficients are visualized in the space of *l* versus *w*.

### Realization of the wideband AWPCS

In order to calculate the local phase shifts required to correct the large aperture phase non-uniformity of the ad hoc horn designed in “Shortened conical horn antenna with larger apex angle”, near-field phase values at 11 GHz were probed on the antenna aperture in cylindrical coordinates with $\phi =0$ and a sampling step-size of $\lambda /3$ chosen based on the unit-cell size explained in “Electromagnetic and mechanical specifications of the proposed unit cell”.

**Figure 3:**
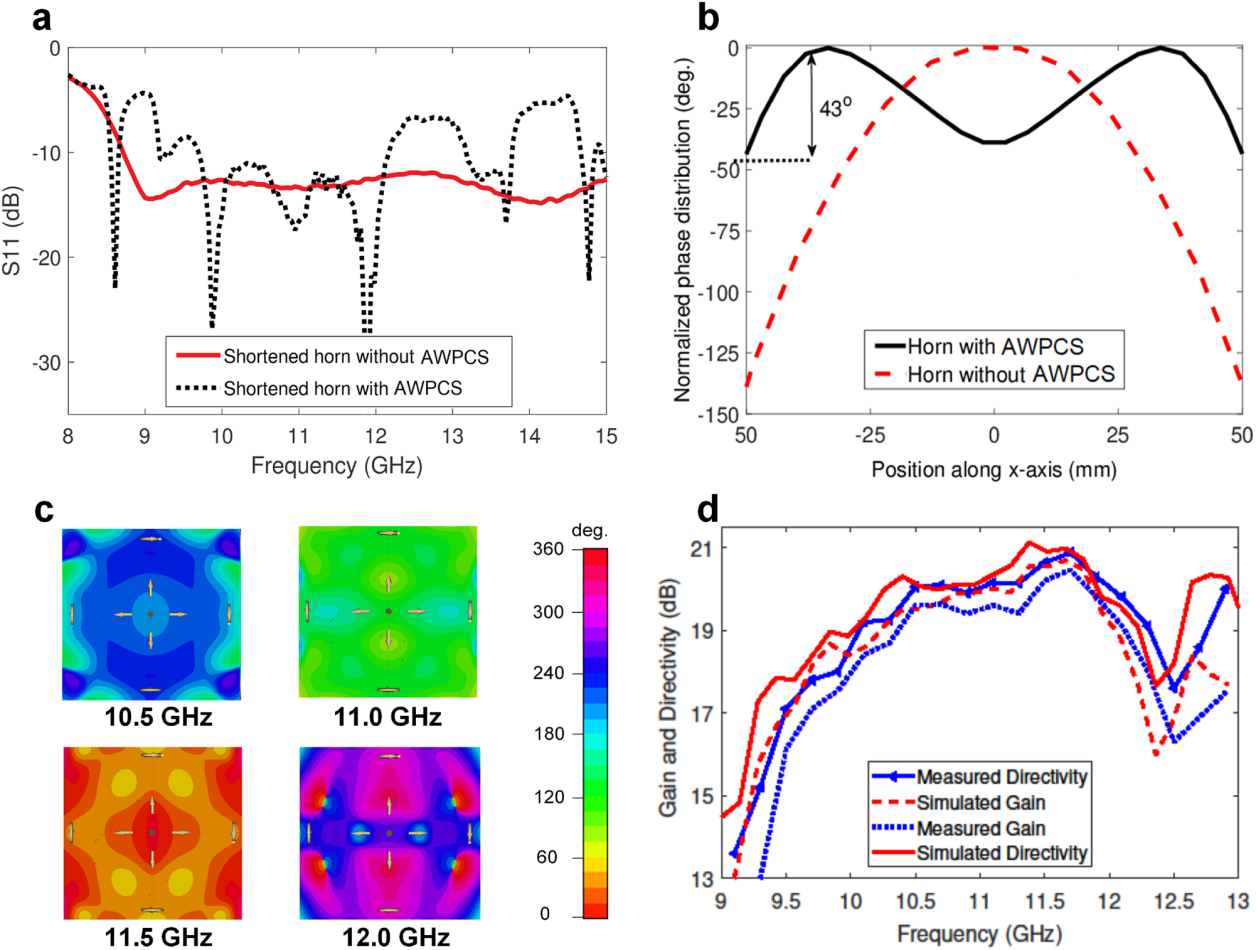
**(a)** Input reflection coefficients of the shortened (ad hoc) horn antenna with and without AWPCS. **(b)** Aperture phase distribution of the shortened horn antenna before and after placing AWPCS. The phase is measured at a distance of 7 mm from the antenna. **(c)** Aperture phase distribution of the shortened horn antenna before and after placing AWPCS. The phase is measured at a distance of 7 mm from the antenna. **(d)** Gain and directivity of the shortened horn antenna with the fully metallic metasurface.

Considering the computed phase non-uniformity (will be shown in dotted red line in Fig. 3b), and the parametric study results shown in surface plots in Fig. 2a,b, it appeared that the DRs with $w= 0.9$ mm and varying length (*l*) can produce most of the required phase-shift values, as depicted in Fig. 2c; however, larger values of *w* can be used for smaller phase delays to complete the required phase range.

In the case of the ad hoc horn antenna, 6 transparent phase-correcting cells with the configuration explained in in “Electromagnetic and mechanical specifications of the proposed unit cell” are required to be distributed on the antenna aperture in a circular arrangement proposed in^10,14^. Such symmetrical manner does not disturb the polarization insensitivity already incorporated in the unit-cell configuration and hence the synthesized AWPCS remains polarization independent. Table 1 shows the size and transmission components of the correcting-phase cells used to synthesize the AWPCS.

In summary, the presented design procedure of the fully metallic spatial phase shifter is summarized into two major steps as follow: Step 1- Designing an electromagnetically transparent sub-structure for the mechanical integrity of the phase shifter. Inductive strips, named Static Cross (SC) are used here for this purpose, as they can be easily tuned to resonate within the desired frequency band of the spatial phase shifter. Step 2- Designing new resonators and attaching them to the inductive strips to control the spatial phase shifter’s transmission phase and magnitude. Any fabricable resonators, such as patch resonators with any shapes, dipole resonators, meander-line resonators, etc. can be added to the sub-structure designed in Step 1 to achieve the desired transmission components. Most of the known resonators are compatible with this design and can be integrated with SCs, thanks to the mechanical stability ensured in Step 1.

**Table 1:** Unit-cell parameters.

Cell	*w*	*l*	$|S_{21}|$	$\angle S_{21}$
	(mm)	(mm)	(dB)	(Deg.)
1	0.9	10.8	− 0.5	190
2	0.9	10.7	− 0.15	201
3	0.9	10.4	− 0.1	225
4	0.9	9.5	− 0.76	259
5	0.9	8.2	− 0.50	285
6	1.9	4.2	− 0.9	345

## AWPCS prototyping

In order to fabricate the proposed AWPCS, firstly the most suitable prototyping procedure needs to be decided based on the constrains of the structure. There are some available fabrication technologies which can be used for metal prototyping, such as Metal Additive Manufacturing (MAM)^45^, plasma cutting^46^, Abrasive Waterjet Cutting (AWC)^47^ and laser cutting^48^, amongst which the first two methods cannot be used for such configurations. Indeed, there is a destructive influence on the confluence, due to the very high temperature used in plasma cutting. Hence this technique is not recommended for the proposed structure, as the perforation areas in the metasurface greatly exceed the untouched areas, which are highly subjected to breakdown. The MAM, however, is capable to precisely 3D-print objects composed of delicate patterns only if the printing object is supported by a robust base which is not the case with our metasurface, due to its small metal thickness (1 mm). Either laser cutting or AWC can be employed to perforate a metal sheet and remove polygonal patterns to realize the proposed metasurface. Nevertheless, laser cutting can be subject to thermal deformation and burr formation when used in a small area. So, a 0.5 Mpsi waterjet cutting machine with garnet abrasive powder was used for this purpose. Figure 2d shows one layer of the AWPCS made of Aluminum. The AWPCS can also be developed by stamping patterns on a thin standard metallic sheet, which can be used for large-scale manufacturing.

Two layers of perforated Aluminum sheets were fabricated and separated by 4 nylon spacers with a hight of 10 mm to realize the wideband AWPCS. The ad hoc horn antenna designed in “Shortened conical horn antenna with larger apex angle” was 3D printed using Original Prusa i3 MK3 3D printer with its maximum infill percentage, and a bed temperature of 110 C. The ad hoc horn antenna was made of polylactic acid (PLA) filament with a minimum layer thickness and a resolution of 0.1 mm and 0.01 mm, respectively, which was then metalized by the copper film. It was finally integrated with the fabricated metasurface to realize the wideband plane-wave horn antenna, as shown in Fig. 2e. The horn antenna is fed by a rectangular slot antenna with dimensions of 15.2 mm$\times$ 8.2 mm.

## Near- and Far-field results

The input reflection coefficients of the bare antenna as well as the antenna under AWPCS loading were measured using an Agilent PNA-X N5242A vector network analyzer. As shown in Fig. 3a, the measured 10-dB |*S*11| bandwidth is $23\%$ with a center frequency of 10.94 GHz.

The predicted near-field results of the antenna with the AWPCS verify a significant improvement in the electric phase distribution throughout a large frequency band, depicted by 1D and 2D plots in Fig. 3b, c, respectively. This near-field correction achieved by the AWPCS has considerably increased the aperture efficiency of the antenna system to 66%, which is 2.7 times grater than that of the bare horn antenna.

**Figure 4:**
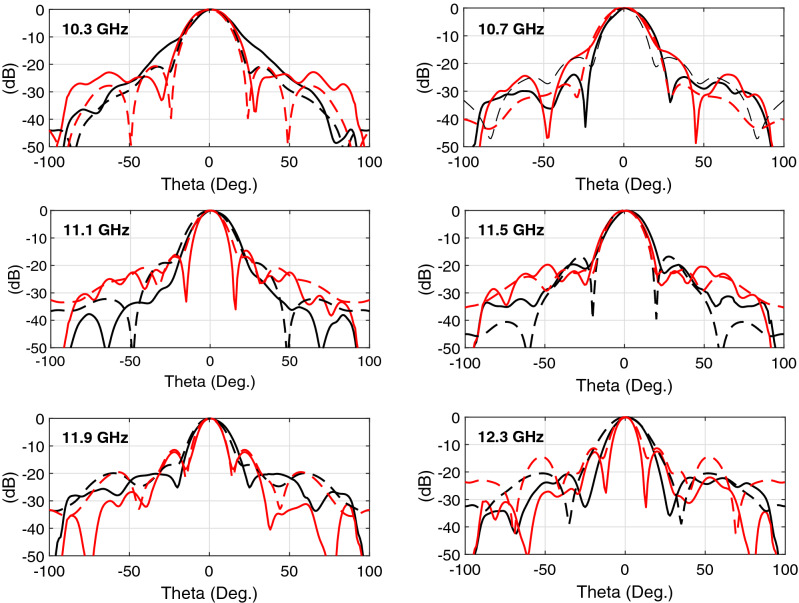
Measured and simulated radiation patterns of the horn antenna with AWPCS at 6 equally spaced frequencies throughout the bandwidth.The solid and dashed lines represent the measured and simulated results, respectively. The black and red lines represent the H- and E-planes, respectively.

In addition to the near-field results, the wideband performance of the AWPCS can be verified through the enhanced far-field results. The plane-wave horn antenna has a measured peak directivity of 20.9 dB with a large 3-dB directivity bandwidth of 25%, extending from 9.70 GHz to 12.45 GHz. The measured peak gain and its corresponding 3-dB bandwidth of the antenna system are 20.46 dB and 23%, which is slightly smaller than the directivity bandwidth. The gain and directivity of the plane-wave horn antenna versus frequency are plotted in Fig. 3d, showing very good agreement between the predicted results by CST MWS and the measured ones in NSI-700s-50 spherical near-field range. The radiation patterns of the antenna under the AWPCS loading at 6 distinct frequencies (10.3, 10.7, 11.1, 11.5, 11.9 and 12.3 GHz) are plotted in Fig. 4, exhibiting very stable sidelobe levels (SLLs) all over the large operating frequency band. As can be seen from Fig. 4, SLLs in the H and E-planes are better than − 20 dB and − 12 dB, respectively, throughout the bandwidth. The weight of the fabricated AWPCS composed of two perforated aluminum sheets is only 30 g. To provide a better overview of the contribution of this work, and underscore the necessity for such a wideband near-field correction achieved by the AWPCS, Table 2 compares some of the state-of-the-art designs with the proposed AWPCS in the aspect of critical specifications for a near-field correction. As reported in this table, the AWPCS has the largest operational bandwidth with a very high aperture efficiency through an extremely low-cost materials and fabrication process.

**Table 2:** Comparison with the recent phase correcting structures.

Publication	No. of microwave substrates	Weight (g)	Peak directivity(dB)	3dB directivity bandwidth	Aperture efficiency	Cost of fabrication
TAP, 2015^8^	8	335	21.6	8%	29%	Very high
TAP, 2016^12^	3	190	20.2	6.4%	26%	Very high
AWPL, 2017^14^	3	193	21.1	11.8%	26%	Very high
MAP, 2017^11^	3	–	20.5	$\approx 3\%$	81.8%	Very high
AWPL, 2018^10^	2	250	22	7%	28%	Very high
TAP, 2020^15^	None	87	20.2	9%	28%	Very low
This work	None	30	20.9	25%	66%	Very low

*TAP* IEEE transactions on antennas and propagation, *AWPL* antennas and wireless propagation letters, *MAP* IET microwaves, antennas & propagation

## Conclusion

An all-metal metasurface, named all-metal wideband phase correcting structure (AWPCS), with a completely passive and symmetrical configuration is presented in this paper. The proposed AWPCS is composed of only two layers of aluminum sheets perforated to realize several arrays of metallic resonators, which are responsible to produce a wideband transmission window with a controllable transmission phase. Unlike all other all-metal phase manipulators, the wideband phase-shifting mechanism of the AWPCS is neither based on polarization conversion, nor sensitive to incoming waves polarization. The proposed AWPCS was fabricated using waterjet cutting machine with abrasive garnet powder to facilitate the perforation of the metal sheets. To test the performance of the AWPCS, a shortened horn antenna with a highly nonuniform near-field phase distribution was used as an electromagnetic source for the AWPCS. According to the predicted and measured results, the AWPCS has greatly improved the near-field phase distribution of the shortened horn antenna, resulting in a very high aperture efficiency of 66%, corresponding to a peak gain of 20.46 dB. The large operational bandwidth of the AWPCS was also verified through the measured large 3-dB gain bandwidth of 23% achieved for the plane-wave horn antenna. The proposed design technology opens a new door in metasurfaces design and their applications, as RF laminates which are the greatest contributor of high manufacturing cost has completely been made redundant without imposing any limitations in terms of electromagnetic behavior, mechanical robustness and ease of fabrication, while an excellent wideband performance has been achieved.
